# Long‐Read, High‐Resolution Sanger Sequencing by Micelle‐Tagging Electrophoresis

**DOI:** 10.1002/elps.70047

**Published:** 2025-10-21

**Authors:** Randall Gamble, H. Michael Wenz, Bashar Mullah, James W. Schneider

**Affiliations:** ^1^ Department of Chemical Engineering Carnegie Mellon University Pittsburgh Pennsylvania USA; ^2^ Thermo Fisher Scientific South San Francisco California USA

**Keywords:** capillary electrophoresis, DNA electrophoresis, end‐labeled free‐solution electrophoresis, Sanger sequencing

## Abstract

We demonstrate a gel‐free electrophoretic separation of Sanger sequencing fragments up to 782 bases in length using nonionic wormlike micelles as drag‐tags in micelle‐tagging electrophoresis (MTE). This is an increase of 280 bases over previous MTE methods and a nearly three‐fold improvement over end‐labeled free‐solution electrophoresis (ELFSE) methods that use covalently attached drag‐tags. For MTE, C18 alkane groups are attached to primers prior to their enzymatic extension. This alkane group provides a binding site for wormlike micelles composed of CiEj‐type nonionic surfactants in the running buffer. Transient attachment of micelles to the C18 alkane group provides a highly uniform drag, equal to that of an ssDNA fragment 309 bases long. To account for slight mobility differences among the BigDye chain terminators, we developed a two‐parameter time‐shifting procedure to align the electropherograms for each termination chemistry. The increase in read length for this low‐viscosity buffer (2.1 cP) is attributed to the alignment procedure, the large yet uniform drag, and the small degree of adsorption‐based band broadening.

## 1 Introduction

Electrophoretic separations of DNA continue to find widespread use in biology labs, forensic analysis, and quality control of plasmids and mRNA used for medical and research purposes. Samples containing fragments that differ by only a nucleotide or two in length require high‐resolution electrophoretic separations. These arise in the analysis of Sanger sequencing products [1], profiling of STR alleles [2], and quantitation of closely related mRNA in multivalent mRNA vaccines. Several gel‐free technologies have been proposed to reduce the runtime of gel electrophoresis, including the use of microfabricated pillar arrays and nanowells in chip‐based platforms. Another of these is end‐labeled free‐solution electrophoresis (ELFSE), which is a rapid, gel‐free electrophoresis method compatible with commercial capillary electrophoresis (CE) instrumentation [3]. ELFSE requires attachment of a negligibly charged drag‐tag to each fragment to bias the fragment mobility (*μ*) in a length‐dependent manner:

(1) $$\mu =\mu_{0}\left(\frac{L}{L+\alpha }\right)$$

Here *μ*_0_ is the mobility of an untagged fragment (approximately length‐nvariant above 20 bases) [4], *L* is the length of the fragment, and *α* is the drag provided by the drag‐tag, expressed as the length of a DNA fragment with equivalent drag. Equation (1) predicts that the mobility difference between fragments will increase with *α*, but at the cost of increased runtime. The resolution of fragments also depends on the peak broadness, which increases with the time allowed for axial diffusion, extent of wall adsorption, and effective widths of injection and detection. In the case of ELFSE methods, polydispersity and chemical degradation of the drag‐tags can also lead to peak broadening [4]. Successful ELFSE separations have required use of small drag‐tags to limit peak broadness arising from tag polydispersity and wall adsorption mechanisms [5, 6, 7, 8, 9, 10, 11].

The resolving power of a DNA separation is characterized by the longest fragment length that can be resolved from a second fragment a single base shorter (“read length”). Read lengths of about 650–900 bases are typical for commercial capillary gel electrophoresis (CGE) systems, with runtimes of 1–3 h depending on the read length desired [1]. Albrecht et al. [12] reported a read length of 280 bases in a 20‐min run using synthetic polypeptides as ELFSE drag‐tags. Longer polypeptides did not improve the read length due to limitations of solid‐phase peptide synthesis, extent of wall adsorption, and loss of polymerase activity due to steric interference of the drag‐tag [4].

Our group has introduced a significant improvement to the ELFSE method that uses nonionic surfactant micelles as drag‐tags [13, 14, 15, 16, 17, 18]. In “micelle‐tagging electrophoresis” (MTE), micelles in the running buffer bind transiently to an 18‐carbon *n*‐alkane group at the 5′ terminus of the DNA fragments to provide a highly uniform drag. The uniform drag is provided by a statistical sampling of different micelle sizes during elution such that each migrating fragment samples a distribution of drags. The mean of that distribution sets the elution time and is highly uniform if the fluctuations are rapid enough to ensure a full sampling of the distribution. We note that this statistical sampling can provide uniform drag even when using populations of micelles that are not inherently monodisperse. We have shown that MTE delivers the largest drag‐tags reported without sacrificing peak sharpness [18]. Further, as the drag‐tag is attached only during the separation, there is no interference with the action of polymerase. The micelle size can be tuned to fit a desired separation, and schemes using multiple micelle sizes to achieve faster runs with high read length have been reported [19, 20].

Recently, we demonstrated that MTE gives read lengths over 500 bases by comparing consecutive electropherograms on samples derived from distinct ddNTPs. However, simultaneous four‐color detection requires a properly sequenced elution of four termination products, each with its own dye terminator. This brings with it a concern that the presence of the individual dye terminators will shift the fragment mobility differently for each terminator chemistry, complicating base‐calling. Here, we report the first true four‐color analysis of Sanger sequencing products using MTE and BigDye chain terminators. We show that use of urea at proper levels in MTE running buffers reduces the impact of wall adsorption on peak broadness, leading to read lengths of 780 bases or more. Finally, we present a simple algorithm to account for dye‐based mobility shifts in this MTE method that requires only two adjustable parameters.

## 2 Materials and Methods

### 2.1 Sanger Sequencing Reactions

Sanger sequencing products were created using the BigDye Terminator v1.1 Cycle Sequencing Kit (Thermo Fisher). A 24‐base primer alkylated with an 18‐carbon tail (5'‐C18‐CGCCAGGGTTTTCCCAGTCACGAC‐3', Thermo Fisher) was extended in the presence of the complementary m13mp18 single‐stranded DNA template (New England Biolabs, Ipswich, MA). Four µL of BDT v1.1 Master Mix, 500 ng of m13mp18 DNA template, and 0.64 µL of 5 µM alkylated primer resuspended in DNA water (Thermo Fisher) were combined in a PCR vial to a final volume of 10 µL. The PCR mixture was vortexed for 15 s and transferred to a 2720 Master Cycler (Thermo Fisher). Sanger reactions were subjected to an initial denaturation step of 95°C for 4 min. Next, 35 cycles consisting of a 95°C denaturation step for 30 s, a 50°C annealing step for 30 s, and a 60°C extension step for 40 s were performed. A final extension step of 60°C for 4 min was performed before allowing the sample to cool to 10°C. The reaction mixture was purified using Centri‐Sep spin columns (Thermo Fisher) per manufacturer's instructions. Two PCR reactions were added to each Centri‐Sep spin column to increase sample signal. After spinning, samples were vacuum centrifuged for 15 min to dry then resuspended using 30 µL of Hi‐Di formamide (Thermo Fisher) and heated to 95°C for 2 min before being snap‐cooled on ice.

### 2.2 Micelle Buffer Preparation

Nonionic surfactants pentaethylene glycol monododecyl ether (C12E5) and pentaethylene glycol monodecyl ether (C10E5) were purchased from Bachem (Torrance, CA). Surfactants were heated briefly to 60°C to enable pipetting. Electrophoresis‐grade urea was purchased from Sigma‐Aldrich and used as received. A 2 mL solution of 62 mM C12E5, 5 mM C10E5, and 7 M urea was prepared in 1× TBE (Sigma‐Aldrich, pH 8.2) and shaken for 1 h. Next, 0.1% v/v POP‐6 polymer (Thermo Fisher) was added to the buffer solution followed by vigorous vortexing for 15 s. The solution was then centrifuged for 15 min at 4000 rpm and transferred to a glass buffer syringe for use in capillary electrophoresis.

### 2.3 Capillary Electrophoresis

MTE was carried out using the ABI Prism 310 Genetic Analyzer (Thermo Fisher). Fused‐silica capillaries (Polymicro Technologies) with 30 µm inner diameter were cut to a total length (*l_c_*) of 43 cm, with a window burned 11 cm from the end, setting the length to detector (*l_d_*) to 32 cm. New capillaries were initially rinsed with 10% v/v POP‐6 solution in 1× TBE for 20 min using the ABI 310 syringe polymer delivery system. In some cases, the initial 10% v/v POP‐6 was incubated for 12 h to further suppress electroosmotic flow. The capillary was then rinsed with the desired micelle surfactant buffer for 8 min. The alkylated Sanger sample was then injected at 2.5 kV for 15 s. Electrophoresis was performed at 15 kV for 30 min at temperatures ranging from 33°C to 44°C. For subsequent runs, the capillary was re‐rinsed using the micelle surfactant buffer for 8 min, and the inlet capillary end and corresponding electrode were dipped three times in deionized water.

## 3 Results and Discussion

We began this study by using a method published by our group [18] that uses a buffer containing a moderate level of urea (3 M) and a binary mixture of CiEj‐type nonionic surfactants (48 mM C12E5; 6 mM C10E5 in 1× TBE, pH 8.2). Although the signal for the T‐terminated channel was high, the signals for the A‐, C‐, and G‐terminated channels were unacceptably low. To more closely match the composition of commercial CGE buffers, we repeated the runs using 7 M urea. This restored the signal in all four channels, but at the expense of decreased micelle size and read length. We then reformulated the buffers to grow longer wormlike micelles by decreasing the concentration of the end‐capping surfactant C10E5 and increasing that of C12E5. A buffer containing 62 mM C12E5, 5 mM C10E5, and 7 M urea in 1× TBE (pH 8.2, *T* = 42°C) gave good signal in all four channels and good resolution of fragments. The elution times for the fragments agreed well with ELFSE theory (Equation 1), which predicts elution time (*t*) for a given fragment length (*L*, in bases) when the tag and DNA are hydrodynamically unsegregated [21]:

(2) $$t=\frac{l_{c}l_{d}}{\mu_{0}V}\left(1+\frac{\alpha }{L}\right)$$

where *l_c_* is the total capillary length, *l_d_* is the length to the detector, *V* is the applied voltage, and *μ*_0_ is the mobility of untagged DNA in free solution, which is not a strong function of *L* for fragments greater than 20 bases [4]. The parameter *α* is a measure of the micelle size, expressed as the length of an ssDNA fragment that would have the same drag as the attached micelle. A plot of elution time versus inverse length (Figure S1) for each of the four channels shows the linearity predicted by Equation (2), with fitted parameters *α* = 309 and *μ*_0_ = 2.05 × 10^−4^ cm^2^/V s. The excellent fit suggests that other electrophoresis mechanisms, such as sieving, are not contributing to the length‐dependent electrophoretic mobility. This *α* value is smaller than observed by Istivan et al. [18] using MTE (*α* = 509) but much larger than observed by Albrecht et al. [12] using covalently attached polypeptide drag‐tags for Sanger sequencing (*α* = 56).

**FIGURE 1:**
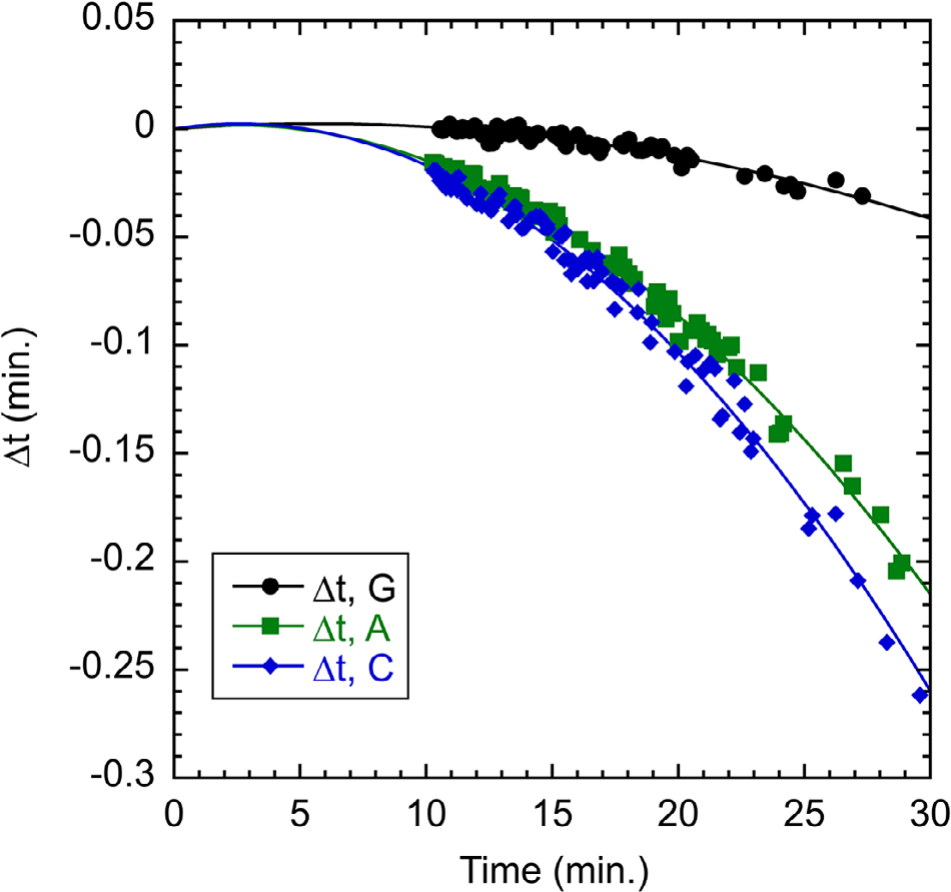
Time shift factors for the G‐, A‐, and C‐terminated channels compared to the T‐terminated channel. For the G‐channel, *a* = 8.513 × 10^−4^ and *b *= −7.496 × 10^−5^ min^−1^, for the A‐channel, *a* = 1.386 × 10^−3^ and *b* = −2.853 × 10^−4^ min^−1^, and for the C‐channel, *a* = 1.804 × 10^−3^ and *b* = −3.489 × 10^−4^ min^−1^, as defined in Equation (3).

There are small mobility differences among the various terminations in Figure S1, and although they are difficult to discern in the plot, they do impact the assignment of fragment lengths to termination chemistry when all four channels are plotted together. Roughly speaking, the G‐ and T‐terminated channels have a similar mobility as do the A‐ and G‐, but these two pairs of channels are slightly displaced from each other. We presume that the shifts are due to slight differences in the mobility of dye terminators, an effect that is also observed in Sanger sequencing using a sieving matrix. The shifts may be further impacted by the binding of surfactant to the dye terminators in MTE. For four‐channel alignment, we calculated shift factors that could be added to the time axis of the A‐, C‐, and G‐terminated channels to bring them in line with the T‐terminated channel. For each fragment length in the sample, we calculated the expected elution time if it were T‐terminated by a linear interpolation of the T‐terminated data in Figure S1. As the T‐channel had the fastest elution, all the A‐, C‐, and G‐channel shift factors were negative in sign. Figure 1 shows a plot of the shift factors obtained by this procedure as a function of elution time, showing a quadratic dependence:

(3) $$t_{\mathrm{shif}t}=\mathrm{at}+bt^{2}$$

where *a* and *b* are fitted parameters for each channel. For alignment, we calculated shift factors for each elution time data point by Equation (3) and added them to time axis for the A‐, C‐, and G‐channels.

**FIGURE 2:**
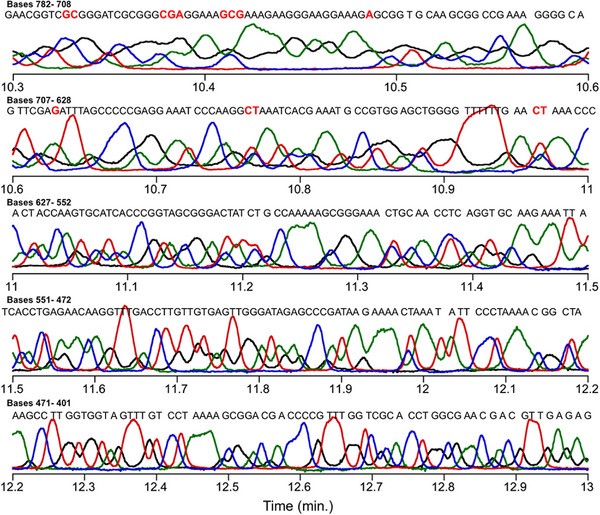
Aligned MTE electropherogram collected using the ABI Prism 310 Genetic Analyzer benchtop capillary electrophoresis instrument. Separation (15 kV, 43 cm capillary) utilized electrokinetic injection (2.5 kV, 15 s) at 42°C and a micelle buffer composed of 62 mM C12E5, 5 mM C10E5, and 7 M urea in 1× TBE. Sanger samples were end‐alkylated using an 18‐carbon, 24‐base primer. T‐terminated products are listed in red, C‐terminated in blue, A‐terminated in green, and G‐terminated in black. The expected sequence is listed above the trace, with red letters indicating incorrect sequence homology. The A‐ and C‐terminated electropherograms have been aligned using a two‐parameter time shift with respect to the T‐terminated channel.

**FIGURE 3:**
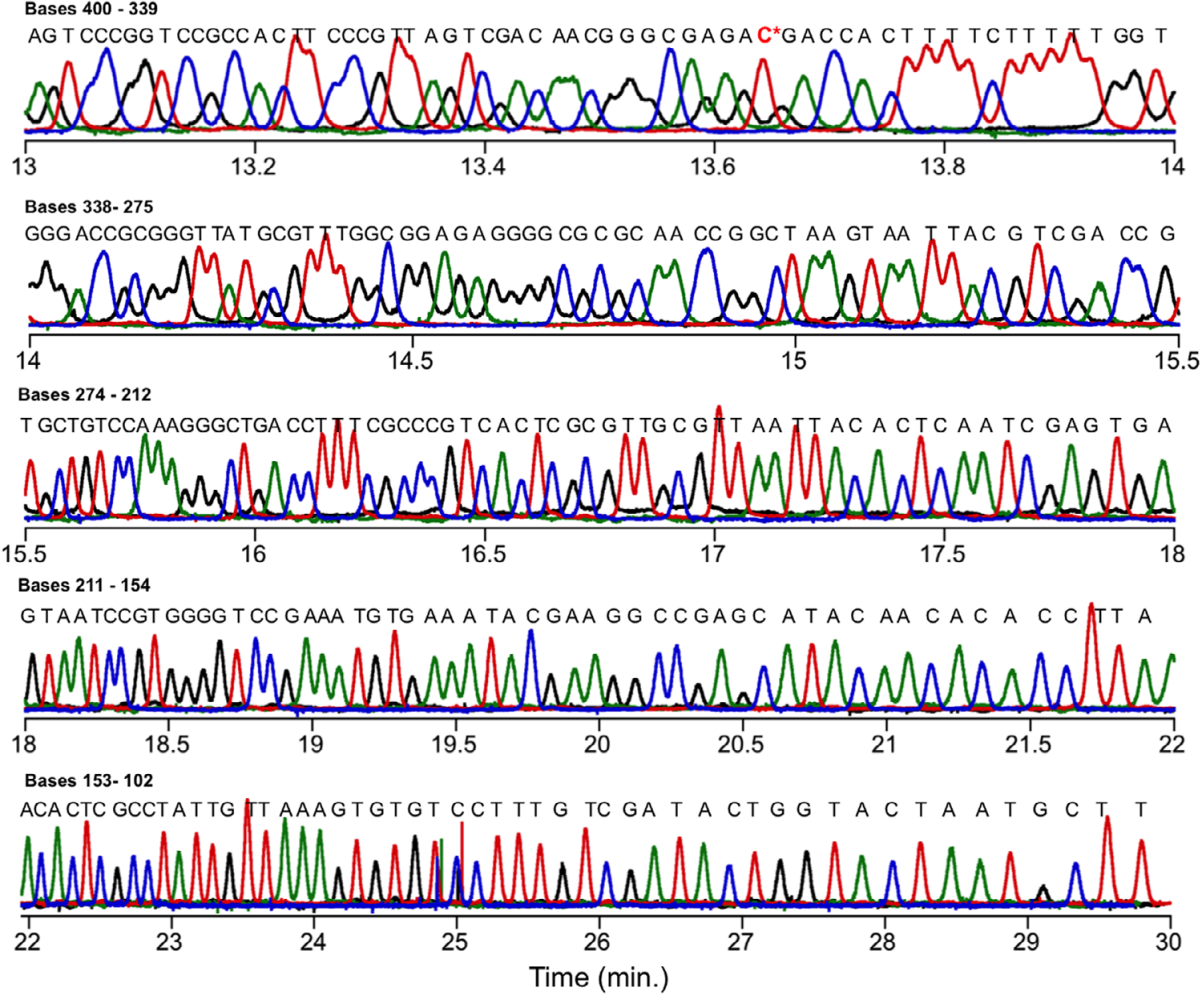
Aligned MTE electropherogram continued from Figure 2, showing elution from *L* = 400–102. Total runtime is 30 min.

This procedure successfully brought the four channels into alignment as shown in Figures 2 and 3. Figure 2 shows the aligned traces for fragments 782–401, eluting between 10.3 and 13.0 min. For determination of read length = 782, we took the longest length that was still read in the proper order based on the sequential elution times of the peak maxima. Still, there were some base‐calling errors in the range of 782–708 involving C‐ and T‐terminated fragments. Beyond *L = *782, the order could no longer be discerned. Figure 3 shows traces for fragment lengths from 400 to 102 bases eluting between 13.0 and 30 min, showing nearly 100% homology with the m13mp18 template. However, the 358 nt fragment was misassigned as T‐terminated when C‐termination was expected. We believe that this is due to a fault in the template, and since it only occurred at this location it was not investigated further.

We also attempted to align the channels using only A‐ and C‐shift factors, and the results were nearly indistinguishable from Figures 2 and 3.This indicates that the use of G‐shift factors is not needed. However, using average *a* and *b* values for A‐ and C‐ to align those channels did lead to a loss of read length (down to 736 bases). Alignment of the four channels using MTE in a general case could likely be accomplished using no more than four fitted parameters to account for differences in dye mobility, although we did not collect *a* and *b* parameters using another template to check for consistency.

In a previous report, we called 502 bases using different instrumentation and buffers with lower levels of urea leading to a large micelle size (*α* = 509) [18]. With this four‐color method, we can now call 782 bases with a much smaller drag‐tag (*α* = 309). In the absence of other band‐broadening effects, resolution and read length are expected to increase with larger *α*. To better understand the dependence on micelle size we calculated the sources of peak broadness by a van Deemter analysis. Here, we obtain the peak resolution (*R*) from the temporal full‐width at half‐maximum (FWHM) and elution time (*t*) of the peaks in the T‐terminated channel as a representation of all channels. As in previous ELFSE work [4], we define *R* as

(4) $$R=\frac{\mathrm{FWHM}}{\left|\partial t/\partial L\right|}$$

where |*∂t*/*∂L*| is the peak spacing per length difference between neighboring fragments, found by differentiating Equation (2). As defined in Equation (4), poorer resolution is indicated by larger values of *R*. We note that other definitions of *R* have the numerator and denominator switched, with an average value taken for FWHM of two neighboring peaks [22]. For Sanger sequencing, the peaks are very closely spaced with nearly equivalent *L* so this averaging is generally not necessary. Use of Equation (4) also allows us to define a theoretical single‐base resolution when fragments a single base longer or shorter in length are not present in a sample. This is accomplished by differentiation of Equation (2) to obtain *∂t/∂L*.

**FIGURE 4:**
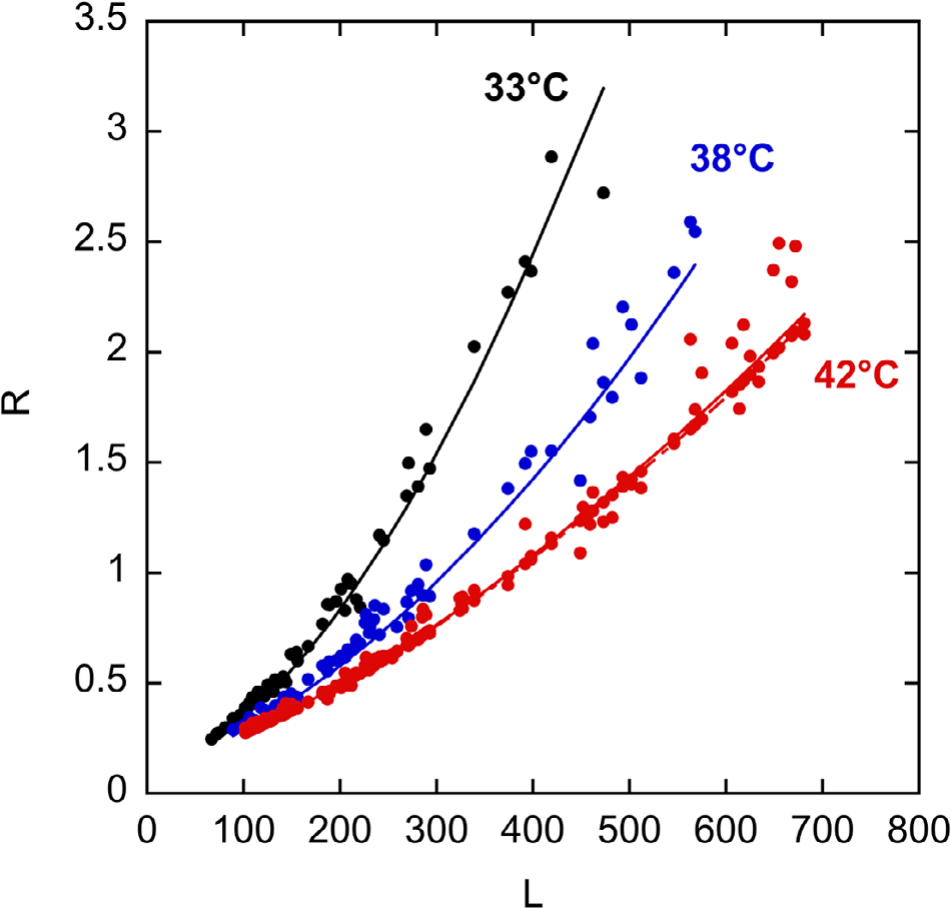
Resolution factor *R* versus DNA fragment length (*L*) at various electrophoresis temperatures by MTE using the 62 mM C12E5, 5 mM C10E5, and 7 M running buffer. Theoretical fits (shown as solid lines) are based on Equation (7) assuming only diffusion (*D*_0_ = 4.4 × 10^−6^ cm^2^/s) and wall adsorption for band broadening, and fitted values for *α* (as shown in Figure S1). A theoretical fit assuming *W *= 0 (shown as a dashed line) is included for the 42°C data and is barely distinguishable from the case where *W* was an adjustable parameter (*W* = 3.2 × 10^−6^ s). 33°C: *α* = 164, *W* = 2.3 × 10^−4^ s; 38°C: *α* = 243, *W* = 7.4 × 10^−5^ s.

Figure 4 plots *R* versus *L*, showing the expected increase in *R* (corresponding to lower resolution) as length increases. The data of Figure 4 can be fit by a modified version of equation (4):

(5) $$[R=\sqrt{8\ln2\;}\left(\frac{L}{\alpha }\right)\left(L+\alpha \right)\sqrt{\frac{H}{l_{d}}}$$

where *H* is the theoretical plate height. Contributions from injection/detection widths, Joule heating, and tag polydispersity are negligible, so *H* is set by diffusion of the tag‐DNA complex along with wall adsorption:

(6) $$H=\frac{2D_{0}t}{l_{d}\sqrt{L+\alpha }}+\frac{Wl_{d}}{t}$$

where *D*_0_ is the monomer diffusion coefficient, which has a value of 4.4 × 10^−6^ cm^2^/s in typical electrophoresis buffers [5], and *α* = 309 as described above. Combining Equation (5) with Equation (6), we have

(7) $$R=\sqrt{8\;\ln\;2}\left(L\right)\left(1+\frac{L}{\alpha }\right){\left[\frac{2D_{0}t}{l_{d}^{2}\sqrt{L+\alpha }}+\frac{W}{t}\right]}^{1/2}$$

which can be fit to the data in Figure 4 (*T* = 42°C), with *W* the only fitting parameter, given fits to *α* and *μ*_0_ as shown in Figure S1. The resulting *W* = 3.2 × 10^−6^ s, which is 100× smaller than the value reported in our previous work [18]. In fact, this fitted *W* is so low that the resolution can be predicted well assuming only diffusive band‐broadening (dashed line, Figure 4). Note that the fit is excellent using the literature value of *D*_0_ = 4.4 × 10^−6^ cm^2^/s without adjustment. We conclude that the large reduction in the wall‐adsorption term is responsible for the higher read lengths despite the smaller micelle size when compared to Istivan et al. [18]. As an added benefit, the smaller drag‐tag also gives faster mobilities and runtimes compared with the previous study. Note also that *R* is about 2.5 at the read length of *L* = 780 bases; this will serve as a benchmark in later discussion.

**FIGURE 5:**
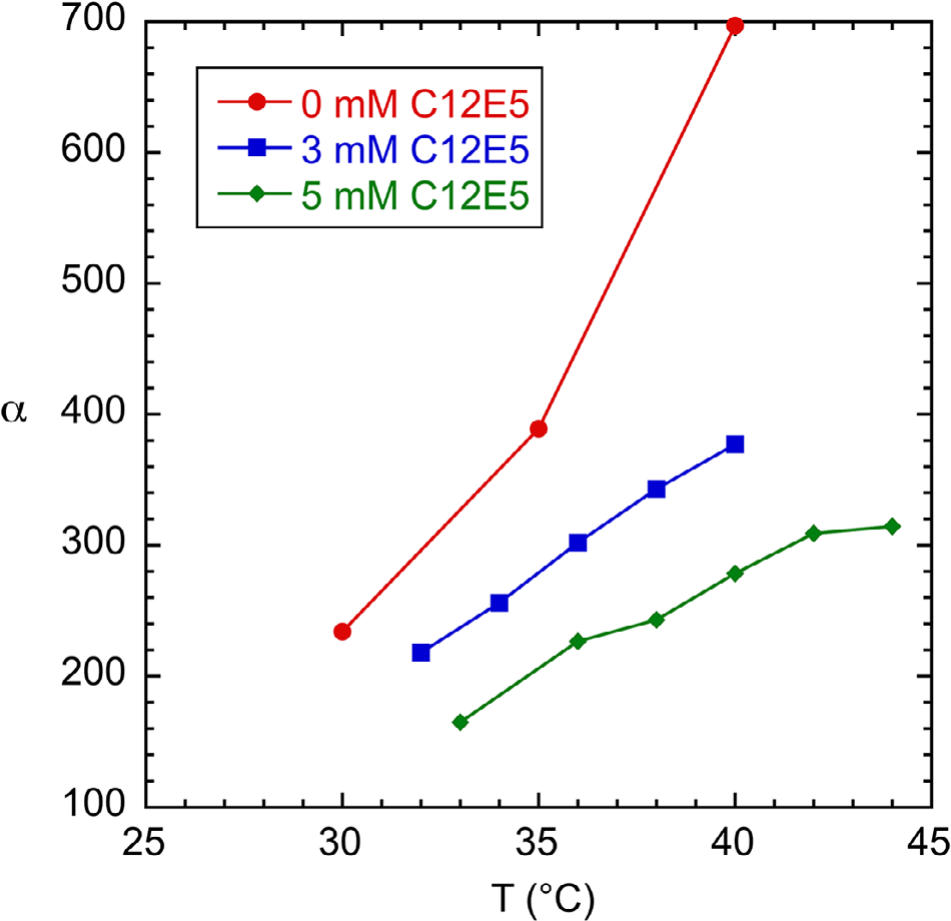
Plot of micelle size (*α*) versus temperature obtained for micelle surfactant buffers with varying C10E5 “end‐capping” concentrations. C12E5 and urea concentrations were set at 62 mM and 7 M, respectively. Note that the micelle sizes increase with temperature and decrease with added end‐capping surfactant. Lines are to guide the eye.

**FIGURE 6:**
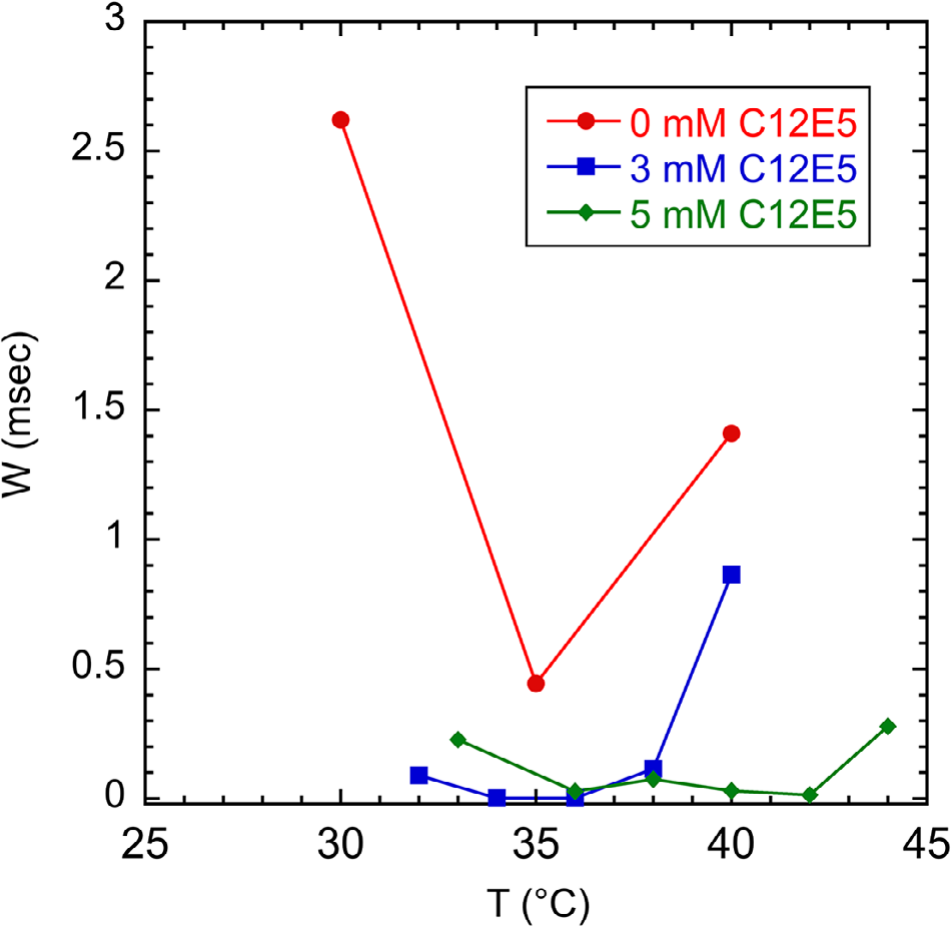
Plot of wall adsorption parameter (*W*) versus temperature for data shown in Figure 5. The lowest wall adsorption value (*W* = 3.2 × 10^−6^ s) occurs at 42°C and 5 mM C10E5. Note that *W* has a minimum value in each case and increases sharply at higher temperature. Lines are to guide the eye.

**FIGURE 7:**
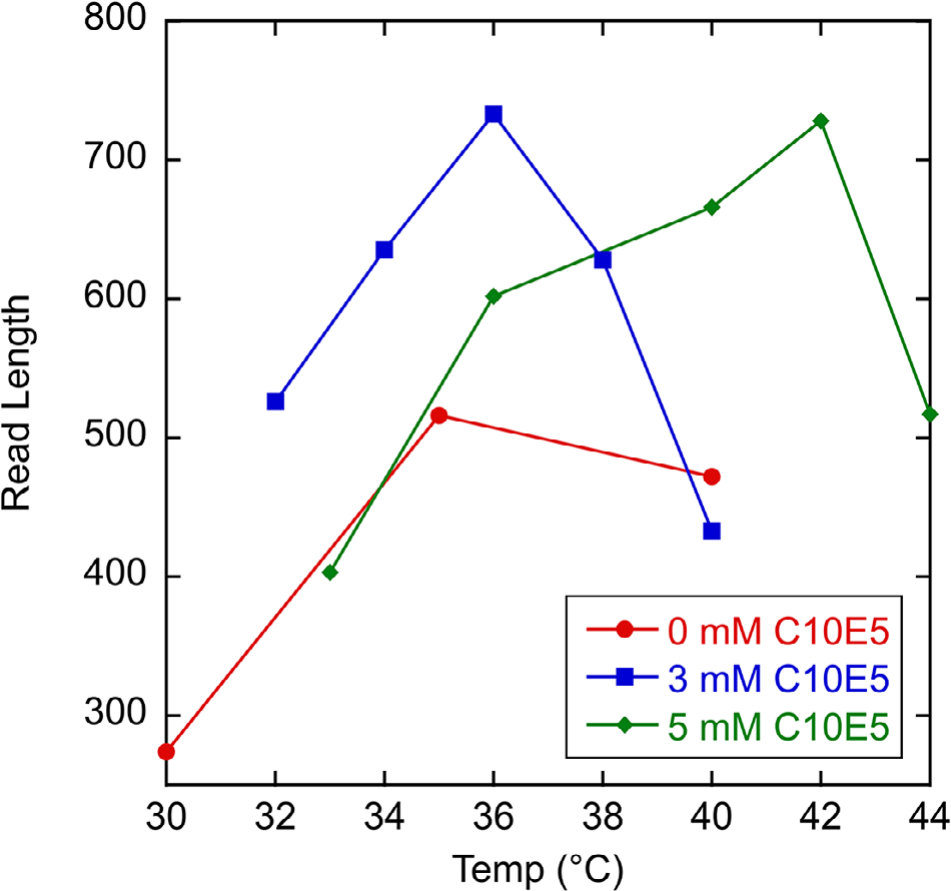
Plot of read length versus temperature for the data shown in Figures 5 and 6. Comparable read lengths are obtained for 3 and 5 mM C10E5 but different optimal temperatures are required to achieve them. Lines are to guide the eye.

Finding an optimal temperature for the MTE‐based Sanger analysis is complicated by two competing effects. As temperature increases, the micelles grow larger, which increases *α* and therefore resolving power and read length. However, as the temperature approaches the cloud point of the surfactant mixture (*T_c_* = 50°C), values for *W* increase abruptly which offsets the higher resolving power of larger micelles. Figures 5 and 6 show the effect of temperature on micelle size (as represented by *α*) and *W*. Addition of urea also has competing effects, acting to reduce micelle size but also reducing *W* as shown in Figures 5 and 6. The combined effect of *α* and *W* on read length is summarized in Figure 7.

As discussed above, we found that the addition of a small amount of end‐capping C10E5 surfactant helped to increase the read length. Figure 5 plots *α* versus temperature for the 62 mM C12E5 and *X* mM C10E5 buffer formulations, where *X* ranges from 0 to 5 mM at a constant urea concentration (7 M). As expected, the micelle size increases with decreasing C10E5 end‐capper and increasing temperature. Higher *α* buffers, however, did not necessarily give the highest read lengths for the 7 M cases. In fact, surfactant buffers containing no C10E5 gave read lengths nearly 300 bases lower than their C10E5‐containing counterparts. Figure 6 plots *W* versus temperature and shows that for buffer formulations containing C10E5, the wall adsorption parameter is much lower and goes through a minimum. It is not clear why this end‐capping surfactant helps reduce *W*.

**FIGURE 8:**
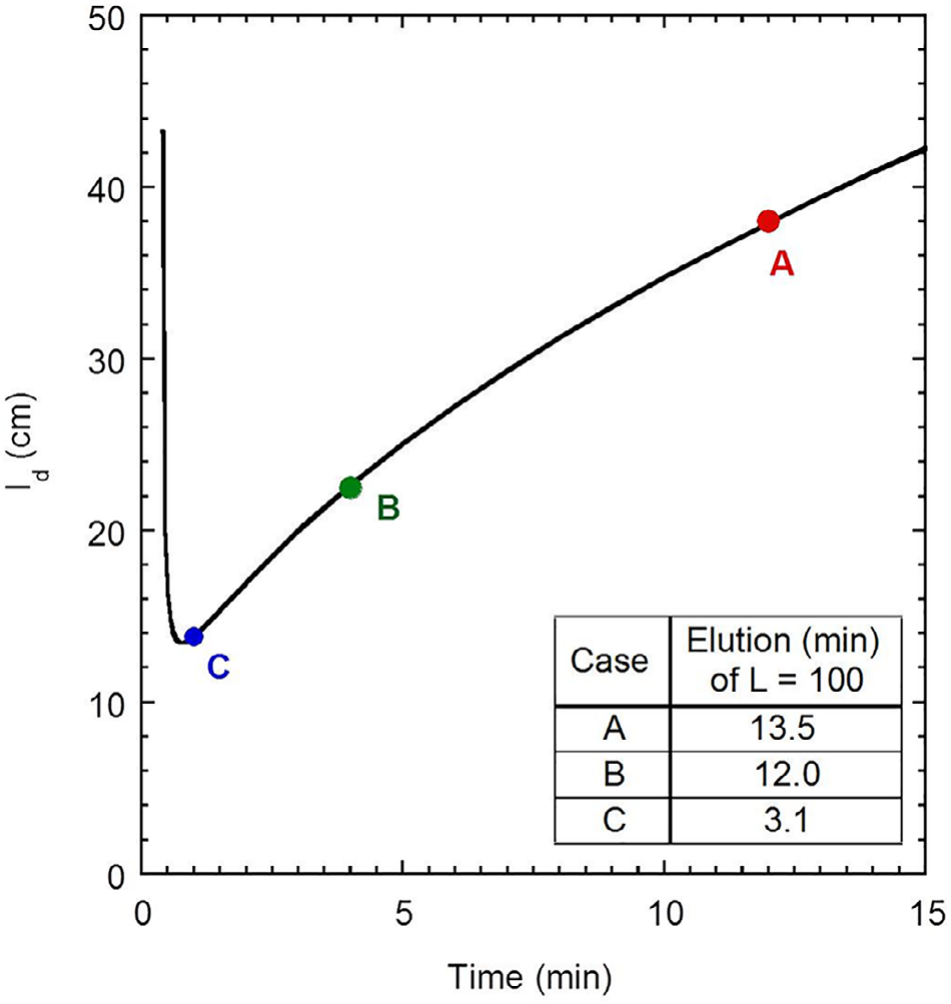
Plot of detector length versus time necessary for a fragment of length 800 to elute with a resolution factor of 2.5, representing the minimum possible run time when imaging the entire capillary length at that time. Scenarios for four‐color CE setups with different electric fields are as follows: A. ABI 310 (*E* = 349 V/cm) used for experimental runs, B. ABI 3130xl (*E* = 606 V/cm) benchtop CE with access to both four‐color and higher electric field, C. Microchip (*E* = 1500 V/cm). The plot is constructed using Equation (7), with *R* = 2.5, *L* = 800, *α* = 309, *W* = 3.2 × 10^−6^ s, and *D*_0_ = 4.4 × 10^−6^ cm^2^/s. The inset shows predicted runtimes for each instrument assuming conventional, “finish‐line” detection and a shortest fragment length of 100 bases.

The ABI 310 system is not ideally configured for fast, high‐read‐length runs by MTE as it is limited by a minimum required capillary length and a maximum voltage. Higher electric fields can be accessed using the ABI 3130xl (maximum voltage = 20 kV, minimum *l_c_* = 22 cm, minimum *l_d_* = 33 cm) or a microchip setup to decrease runtime. If we assume that band‐broadening in MTE is purely diffusive in nature, as suggested above, we can predict optimal read lengths and runtimes for these systems. Here, we will assume a target read length of *L* = 800, *α* = 309, *W* = 3.2 × 10^−6^ s, *D*_0_ = 4.4 × 10^−6^ cm^2^/s, and *R* = 2.5 so that the only unknowns in Equation (7) are length to detector (*l_d_*) and elution time (*t*). Figure 8 plots these parameters and shows a minimum elution time of a barely resolved *L *= 800 fragment to be about 1 min using instrumentation with *l_d_* = 14 cm. Using Equation (2) (with above parameters and *l_c_* = *l_d_*), we find that an electric field of *E = *1600 V/cm would be needed to achieve this elution time., requiring an applied voltage of 22.4 kV. This value is accessible using commercially available power supplies. Assuming operation at maximum voltage, the elution time of base 800 for the ABI 3130xl is 4.5 min.

We emphasize that these are only the times for the *L* = 800 fragment to migrate past a detector some distance away from an injection source. For such a stationary “finish‐line” detector, additional time would be required for the shortest fragment of interest to cross the detector (*L* = 100 in this study). By Equation (2), the ABI 3130xl would require 12.0 min for a full 800–100 nt runtime, and the microchip with *l_d_* = 14 cm would require 3.1 min for 800–100 nt elution. This suggests that systems with shorter columns and higher voltage could drastically reduce runtime compared to the 30 min runtime shown here if band broadening remains set by diffusion alone [2]. Runtimes can be further decreased by imaging the entire channel at once [23], provided that all lengths are resolved at this time. For ELFSE‐type DNA separations, McCormick and Slater [24] have shown that this condition is met once the longest fragments are minimally resolved at the column exit. This imaging format would set the total runtime to the time required for *L* = 800 to elute with minimal resolution, bringing total runtime down to 1.0 min for a typical microchip setup such as those proposed by Le Roux et al. [25, 26] for STR allele analysis.

In addition to the increase in signal, added urea may also improve resolution in MTE. Using sieving polymer solutions, Rosenblum et al. showed that added urea was necessary for acceptable resolution of an ssDNA ladder [27]. Later, Nock et al. [22] observed that added urea improved resolution of STR loci and ascribed it to hydrogen bonding of urea to DNA, increasing the mass of the fragments. They also pointed to improved sample focusing during injection owing to the increased buffer viscosity with added urea. In the case of MTE, urea also impacts the self‐assembly properties of nonionic surfactants [28], and it is difficult to determine the impact of each of these mechanisms on resolution. We note that the free‐solution mobility *μ*_0_ = 2.05 × 10^−4^ cm^2^/V s (Figure S1) is lower than that for typical micelle‐free running buffers (*μ*_0_ ∼ 3 × 10^−4^ cm^2^/V s) [4], as expected given the impact of urea and surfactants on the viscosity of the MTE running buffer.

To check for repeatability, we performed two MTE runs at 40°C (Figure S2) and observed a slightly lower read length (*L *= 666–631) for the second run using the *R* = 2.5 criterion. It is not clear whether this is due to slight changes in the capillary conditioning or drift in buffer properties, or both. Best results were obtained when the capillary was incubated with the POP‐6 coating for several hours prior to use and periodically refilled with POP‐6 during use. We expect that more robust, covalent modifications to the capillary may reduce the need for these treatments and may also improve reproducibility.

In this report, we have presumed *α* to be constant, and as such, we have neglected fragment‐length‐dependent shifts in *α* such as steric segregation [21] and electrophoretic “end‐effects” [29]. As shown in Figure S1, the fragment mobilities agree very well with Equation (2) over a wide range of fragment lengths. We suspect that the use of somewhat smaller micelles and fragments greater than *L* = 100 places the runs in regimes where these effects are not significant.

## 4 Conclusion

We have demonstrated the first true four‐color Sanger sequencing run by MTE with a read length of 782 bases. This is a 497‐base increase in read length compared to previously reported four‐color ELFSE runs, and a 280‐base improvement over previous MTE reports done without true four‐color readout. This increase in read length is attributed to a negligible wall adsorption band‐broadening term (*W* = 3.2 × 10^−6^ s) associated with the 7 M urea surfactant buffer, and is more successful despite a smaller micelle drag‐tag size compared to previous reports (*α *= 309). An alignment procedure was developed for four‐color base‐calling by a simple time shift procedure with minimal fitted parameters, resulting in the final read length of 782 bases. Alternative schemes using electroosmotic flow [19, 20] or high‐field microchip formats can be utilized to substantially decrease runtime without sacrificing read length. We believe that this fast, inexpensive MTE technique can be used in place of gels for many applications such as STR analysis [2] and miRNA detection [15, 16].

## Conflicts of Interest

The authors declare no conflicts of interest.

## Supporting information




**Supporting File 1**: elps70047‐sup‐0001‐SuppMat.docx.

**Supporting File 2**: elps70047‐sup‐0002‐FigureS1.tiff.

**Supporting File 3**: elps70047‐sup‐0002‐FigureS2.tiff.

## Data Availability

Data available on request from authors.

## References

[1] M. M. Detwiler , T. J. Hamp , and A. L. Kazim , “DNA Sequencing Using the Liquid Polymer POP‐7™ on an ABI P Rism® 3100 Genetic Analyzer,” Biotechniques 36 (2004): 932–933, 10.2144/04366BM01. 15211740

[2] J. M. Butler , E. Buel , F. Crivellente , and B. R. McCord , “Forensic DNA Typing by Capillary Electrophoresis Using the ABI Prism 310 and 3100 Genetic Analyzers for STR Analysis,” Electrophoresis 25 (2004): 1397–1412, 10.1002/elps.200305822. 15188225

[3] P. Mayer , G. W. Slater , and G. Drouin , “Theory of DNA Sequencing Using Free‐Solution Electrophoresis of Protein‐DNA Complexes,” Analytical Chemistry 66 (1994): 1777–1780, 10.1021/ac00082a029.

[4] R. J. Meagher , J.‐I. Won , L. C. McCormick , et al., “End‐Labeled Free‐Solution Electrophoresis of DNA,” Electrophoresis 26 (2005): 331–350, 10.1002/elps.200410219. 15657881

[5] H. Ren , A. E. Karger , F. Oaks , S. Menchen , G. W. Slater , and G. Drouin , “Separating DNA Sequencing Fragments Without a Sieving Matrix,” Electrophoresis 20 (1999): 2501–2509, 10.1002/(SICI)1522-2683(19990801)20:12<2501::AID-ELPS2501>3.0.CO;2-H. 10499343

[6] W. N. Vreeland , C. Desruisseaux , A. E. Karger , G. Drouin , G. W. Slater , and A. E. Barron , “Molar Mass Profiling of Synthetic Polymers by Free‐Solution Capillary Electrophoresis of DNA‐Polymer Conjugates,” Analytical Chemistry 73 (2001): 1795–1803. 11338593 10.1021/ac001380+

[7] W. N. Vreeland , G. W. Slater , and A. E. Barron , “Profiling Solid‐Phase Synthesis Products by Free‐Solution Conjugate Capillary Electrophoresis,” Bioconjugate Chemistry 13 (2002): 663–670, 10.1021/bc0155871. 12009959

[8] W. N. Vreeland , R. J. Meagher , and A. E. Barron , “Multiplexed, High‐Throughput Genotyping by Single‐Base Extension and End‐Labeled Free‐Solution Electrophoresis,” Analytical Chemistry 74 (2002): 4328–4333, 10.1021/ac0258094. 12236339

[9] J. I. Won , R. J. Meagher , and A. E. Barron , “Protein Polymer Drag‐Tags for DNA Separations by End‐Labeled Free‐Solution Electrophoresis,” Electrophoresis 26 (2005): 2138–2148, 10.1002/elps.200410042. 15880624

[10] R. J. Meagher , L. C. McCormick , R. D. Haynes , et al., “Free‐Solution Electrophoresis of DNA Modified With Drag‐Tags at Both Ends,” Electrophoresis 27 (2006): 1702–1712, 10.1002/elps.200500554. 16645947

[11] R. D. Haynes , R. J. Meagher , J. I. Won , F. M. Bogdan , and A. E. Barron , “Comblike, Monodisperse Polypeptoid Drag‐Tags for DNA Separations by End‐Labeled Free‐Solution Electrophoresis (ELFSE),” Bioconjugate Chemistry 16 (2005): 929–938, 10.1021/bc0496915. 16029034

[12] J. C. Albrecht , J. S. Lin , and A. E. Barron , “A 265‐Base DNA Sequencing Read by Capillary Electrophoresis With No Separation Matrix,” Analytical Chemistry 83 (2011): 509–515, 10.1021/ac102188p. 21182303 PMC3271724

[13] S. T. Grosser , J. M. Savard , and J. W. Schneider , Analytical Chemistry 79(2007): 9513–9515, 10.1021/ac7016376. 18020426 PMC4504436

[14] J. M. Savard , S. T. Grosser , and J. W. Schneider , Electrophoresis 29 (2008): 2779–2789, 10.1002/elps.200700580. 18546164

[15] J. M. Goldman , L. A. Zhang , A. Manna , B. A. Armitage , D. H. Ly , and J. W. Schneider , Biomacromolecules 14 (2013): 2253–2261, 10.1021/bm400388a. 23777445

[16] J. M. Goldman , S. Kim , S. Narburgh , B. A. Armitage , and J. W. Schneider , Biopolymers 113 (2022): e23479, 10.1002/bip.23479. 34643943

[17] K. Hui , L. Yan , and J. W. Schneider , “Electrophoretically Snagging Viral Genomes in Wormlike Micelle Networks Using Peptide Nucleic Acid Amphiphiles and dsDNA Oligomers,” Biomacromolecules 25 (2024): 4891–4897, 10.1021/acs.biomac.4c00332. 39017713 PMC11322999

[18] S. B. Istivan , D. K. Bishop , A. L. Jones , S. T. Grosser , and J. W. Schneider , Analytical Chemistry 87 (2015): 11433–11440, 10.1021/acs.analchem.5b02931. 26455271

[19] M. A. Fahrenkopf , B. E. Ydstie , T. Mukherjee , and J. W. Schneider , Computers & Chemical Engineering 64 (2014): 63–70, 10.1016/j.compchemeng.2014.01.012. 24764606 PMC3992867

[20] M. A. Fahrenkopf , T. Mukherjee , B. E. Ydstie , and J. W. Schneider , Electrophoresis 35 (2014), 3408–3414, 10.1002/elps.201400266. 25154385 PMC4504435

[21] C. Desruisseaux , D. Long , G. Drouin , and G. W. Slater , “Electrophoresis of Composite Molecular Objects. 1. Relation Between Friction, Charge, and Ionic Strength in Free Solution,” Macromolecules 34 (2001): 44–52, 10.1021/ma0002702.

[22] T. Nock , J. Dove , B. McCord , and D. Mao , “Temperature and pH Studies of Short Tandem Repeat Systems Using Capillary Electrophoresis At Elevated pH,” Electrophoresis 22 (2001): 755–762, 10.1002/1522-2683(200102)22:4<755::AID-ELPS755>3.0.CO;2-S. 11296931

[23] A. Li , X. Chen , and V. M. Ugaz , “Miniaturized System for Rapid Field Inversion Gel Electrophoresis of DNA With Real‐Time Whole‐Gel Detection,” Analytical Chemistry 82 (2010): 1831–1837, 10.1021/ac902490e. 20148578

[24] L. C. McCormick and G. W. Slater , “A Theoretical Study of the Possible Use of Electroosmotic Flow to Extend the Read Length of DNA Sequencing by End‐Labeled Free Solution Electrophoresis,” Electrophoresis 27 (2006): 1693–1701, 10.1002/elps.200500573. 16568501

[25] D. Le Roux , B. E. Root , C. R. Reedy , et al., “DNA Analysis Using an Integrated Microchip for Multiplex PCR Amplification and Electrophoresis for Reference Samples,” Analytical Chemistry 86 (2014): 8192–8199, 10.1021/ac501666b. 25091472

[26] D. Le Roux , B. E. Root , J. A. Hickey , et al., “An Integrated Sample‐in‐Answer‐Out Microfluidic Chip for Rapid Human Identification by STR Analysis,” Lab on a Chip 14 (2014): 4415–4425, 10.1039/C4LC00685B. 25248520

[27] B. B. Rosenblum , F. Oaks , S. Menchen , and B. Johnson , “Improved Single‐Strand DNA Sizing Accuracy in Capillary Electrophoresis,” Nucleic Acids Research 25 (1997): 3925–3929, 10.1093/nar/25.19.3925. 9380518 PMC146964

[28] C. L. Bianco , C. S. Schneider , M. Santonicola , A. M. Lenhoff , and E. W. Kaler , “Effects of Urea on the Microstructure and Phase Behavior of Aqueous Solutions of Poly(Oxyethylene) Surfactants,” Industrial & Engineering Chemistry Research 50 (2011): 85–96, 10.1021/ie101011v. PMC3045059 21359094

[29] L. C. McCormick and G. W. Slater , “The Molecular End Effect and Its Critical Impact on the Behavior of Charged‐Uncharged Polymer Conjugates During Free‐Solution Electrophoresis,” Electrophoresis 26 (2005): 1659–1667, 10.1002/elps.200410276. 15812840

