# Supplementary material for: Long‐Read, High‐Resolution Sanger Sequencing by Micelle‐Tagging Electrophoresis
Source: Electrophoresis. 2025 Oct 21;47(1):5–12. doi: 10.1002/elps.70047 (PMC12827225; doi:10.1002/elps.70047)
Supplement: Supplementary file 1 — Supporting File 1: elps70047‐sup‐0001‐SuppMat.docx. [file ELPS-47--s003.docx]

**Supplemental Information**

**
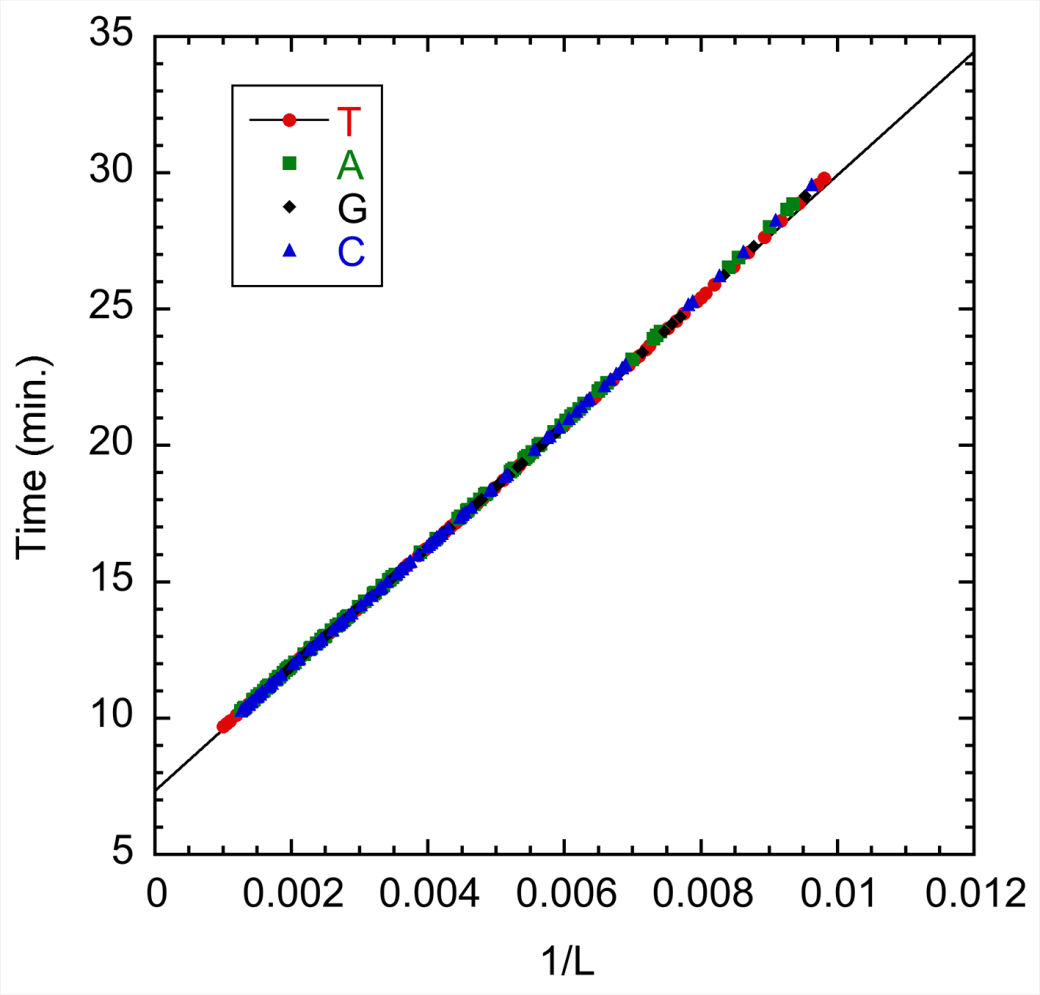
**

**Figure S1.**

DNA fragment elution time versus inverse DNA length (1/*L*) for the 62 mM C12E5, 5 mM C10E5, and 7 M urea at 42°C; *α* = 309, *µ_o_* = 2.05 x 10^-4^ cm^2^/V-s. Fit is to eq (2) showing that assumption of constant *α* is valid.

**Figure S2.** Resolution factor *R* versus DNA fragment length (*L*) for repeated MTE runs using the 62 mM C12E5, 5 mM C10E5, and 7 M running buffer at 40°C. Theoretical fits (shown as solid lines) are based on eq 6 assuming only diffusion and wall adsorption for band broadening and fitted values for *α*. Using *R* = 2.5 as an approximate criterion for read length, the read length for the first run (blue) was *L* = 666 and that for the second run (red) was *L* = 631.
