# Supplementary figures and images for: Long‐Read, High‐Resolution Sanger Sequencing by Micelle‐Tagging Electrophoresis
Source: Electrophoresis. 2025 Oct 21;47(1):5–12. doi: 10.1002/elps.70047 (PMC12827225; doi:10.1002/elps.70047)

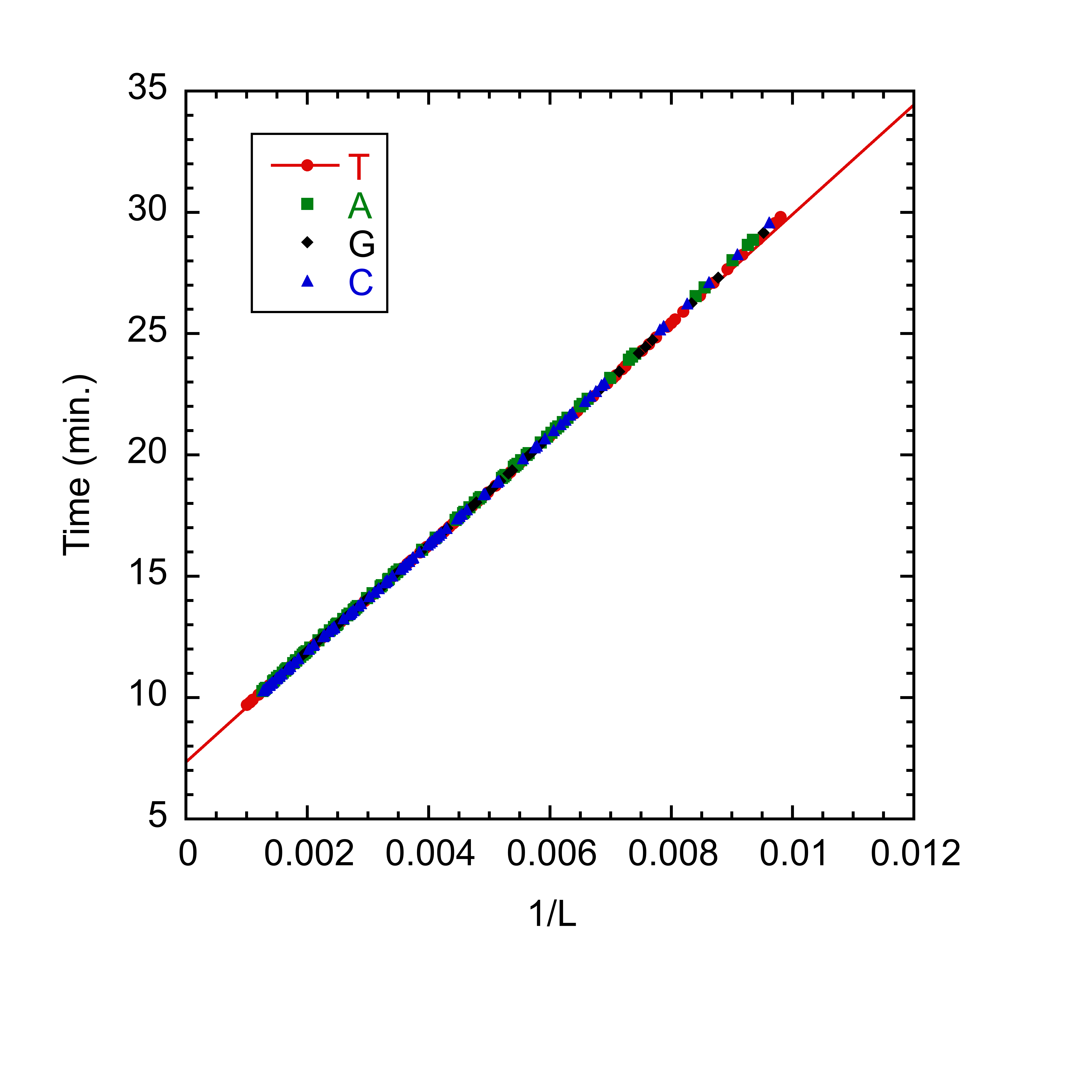

Supplement: Supplementary file 2 — Supporting File 2: elps70047‐sup‐0002‐FigureS1.tiff. [file ELPS-47--s001.tiff]

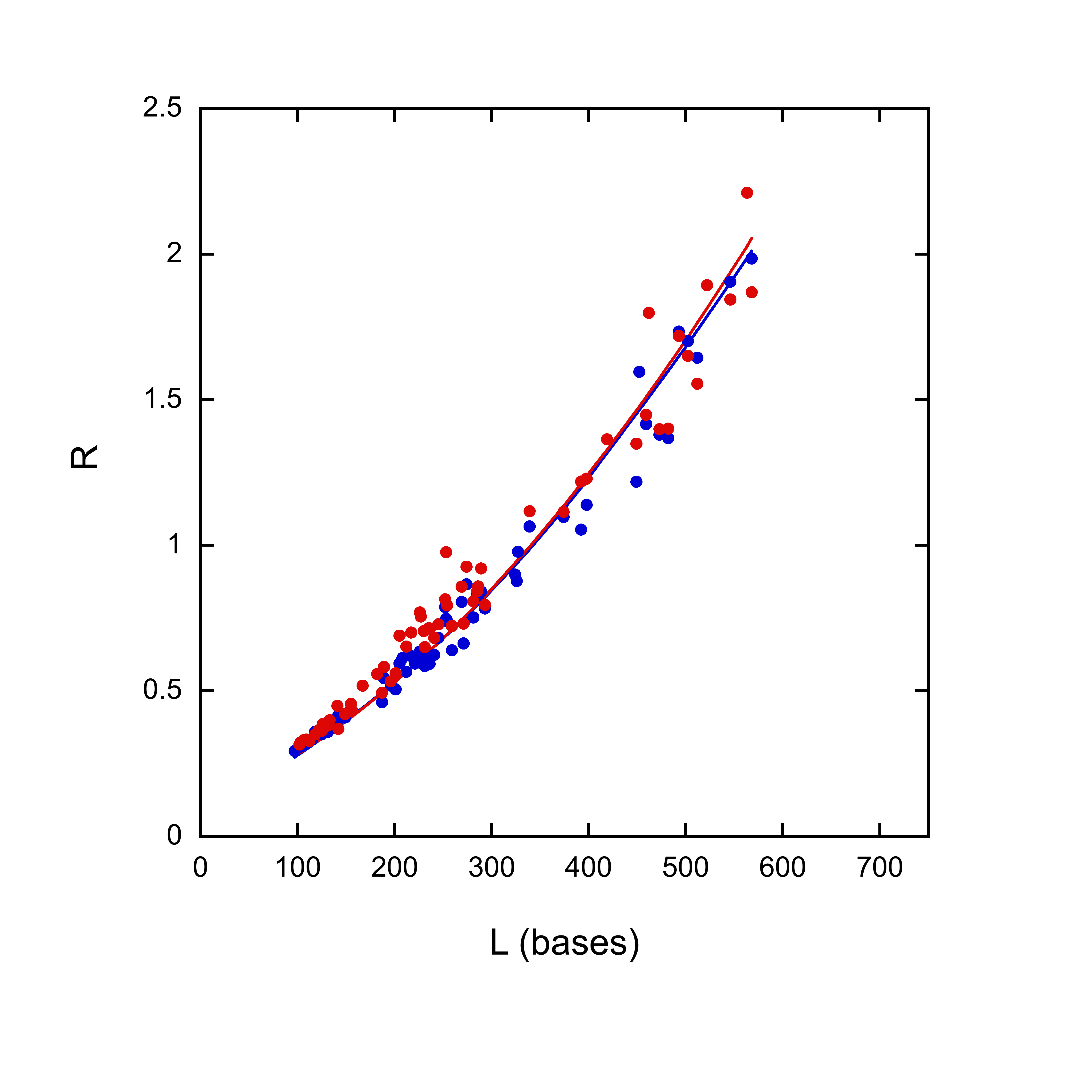

Supplement: Supplementary file 3 — Supporting File 3: elps70047‐sup‐0002‐FigureS2.tiff. [file ELPS-47--s002.tiff]
